# Sale and Purchase of a Medical Practice

**Published:** 1899-11-04

**Authors:** 


### The Institutional Workshop.

### SALE AND PURCHASE OF A MEDICAL PRACTICE.

At some stage of his existence the medical practitioner will probably have to consider the sale or purchase of a practice. Whether he be vendor or purchaser, it is useful that he should know the nature of the duties and responsibilities which follow upon such a transaction, and the restrictions which, if he be vendor, he places himself under with regard to subsequent practice in the neighbourhood.

In its very nature the sale of a medical practice involves the execution of a contract which is against the policy of English law—that is to say, it is an agreement in restraint of trade, and is therefore contrary to public policy. If a man deliberately sells the right which all men have in a free country to the exercise of their calling, he deprives the public of the benefit of his skill and experience, while he abandons what may be his means of livelihood. A principle of law has therefore been evolved, having its origin in the famous case of Mitchell v. Reynolds, which was decided in 1712, that contracts in restraint of trade are void and must not be enforced by the Courts. If a medical man at the present day were to enter into a bond which prevented him from practising anywhere for the rest of his natural life, no court of justice would enforce its due performance.

While, therefore, contracts in total restraint of trade are void, it has become a well-settled rule that if restriction is limited as to area, and there is "consideration" for the bargain, the good-will of a medical practice or of a business may be made the proper subject-matter of a contract. And so the well-known "bond" into which the vendor of a practice enters is recognised in, and will always be enforced and respected by, a Court of Justice. Were it otherwise the sale of a connection would be rendered impossible, as nothing could operate to prevent the outgoing practitioner from interfering with the practice which he himself had disposed of for a substantial sum of money.

Such is the bare outline of a branch of law which is surrounded with many technicalities. Into these it is not our purpose to enquire at any length, but there are a few points which demand a further elucidation. It has been pointed out that the restriction must be limited as to space. It would be wholly unreasonable, for instance, to prevent the vendor of a practice from exercising his calling anywhere in the British Isles. Thus a general practitioner in London could not interfere with a Liverpool connection, nor could a practice in Scotland be in any way prejudicial to an Irish business. Many cases have arisen in the Courts as to what is a reasonable limit. In Horner v. Graves (7 Bing. 735) it was held to be unreasonable to restrict a dentist from practising anywhere within 200 miles from York. On the other hand, to borrow an instance from the legal profession, a covenant preventing a solicitor from practising as such anywhere in England for 20 years has been held to be reasonable and capable of enforcement (Whittaker v. Howe, 3 Bras. 383). It follows, therefore, that in marking the border line, the Court will be influenced in a large measure by the nature of the business. The available means of communication are also important. By means of railways, telephones, &c., a practice can be extended over a much wider area at the present day than would have been possible at the close of last century. That the Court is willing to enlarge the boundary in certain cases, even to the confines of the habitable globe, was clearly demonstrated in 1896, when world-wide restriction was placed upon the manufacture of a particular class of guns and armament. Although this case could never have any direct application to the medical profession, yet it serves to show the elastic nature of rules by which the Court is governed in these matters.

With regard to time there does not appear to be any restriction. Nevertheless if a man binds himself not to practise for a limited period the area of restriction may receive a slight extension. The rule by which the boundary should be determined was thus expressed in the case of Horner v. Graves, to which allusion has already been made :—

"The restraint must be only such as to afford a fair protection to the interests of the party in favour of whom it is given, and not so large as to interfere with the interests of the public."

The consideration—another essential feature of these contracts—is the next matter which deserves attention. And upon this point, so long as the Court is satisfied that there is a consideration which is not merely colourable, the contract will be enforced. The adequacy of the consideration is left entirely to the parties themselves. It is not surprising that the Court refuses to interfere, for how could any tribunal decide what is a reasonable price to pay for a medical practice? Instances could be cited without number to show what has been held a reasonable consideration. In the case of Sainter v. Fergusson (7 C.B. 716) the engagement of the defendant as assistant was held to be sufficient consideration for an undertaking on his part not to practise within a radius of seven miles after the termination of his employment.

Having made an attempt to indicate the features which are essential to contracts in restraint of trade, and, therefore, to contracts for the sale of a medical practice, we now propose to consider what constitutes a breach of such a contract. In most contracts the covenant is in the following form: The vendor undertakes that he "will not at any time hereafter, directly or indirectly, and either alone or in partnership with or as assistant to any other person or persons carry on the profession or business of a surgeon or physician" within a certain area. A further clause prohibiting practice "in open competition" sometimes finds its way into the deed.

The question naturally arises, What constitutes practising? Most medical men—or their consciences—will be able to answer this question without any difficulty, but doubtful points arise from time to time. In the case of Palmer v. Mallett (36 Ch., D. 411) it was decided that a person acting only as a salaried assistant was carrying on the profession of a surgeon, and that to do so was a breach of the covenant. The words "open competition" were discussed in the case of Rogers v. Drury (57 L. J., Ch. 504(. There the vendor agreed not to practise within a radius, or directly or indirectly compete with the purchaser. He visited certain patients within the prohibited area at their own request. These patients, moreover, had refused to call in the purchaser on any account. This was held to constitute an infringement of the covenant, which the Court said was not confined to active competition.

